# Approaches to Enhance Sugar Content in Foods: Is the Date Palm Fruit a Natural Alternative to Sweeteners?

**DOI:** 10.3390/foods13010129

**Published:** 2023-12-29

**Authors:** Estrella Sayas-Barberá, Concepción Paredes, Manuel Salgado-Ramos, Noelia Pallarés, Emilia Ferrer, Casilda Navarro-Rodríguez de Vera, José Ángel Pérez-Álvarez

**Affiliations:** 1 Instituto de Investigación en Innovación Agroalimentaria y Agroambiental (CIAGRO-UMH), Miguel Hernández University, EPS-Orihuela, Ctra. Beniel km 3.2, 03312 Orihuela, Alicante, Spain; estrella.sayas@umh.es (E.S.-B.); c.paredes@umh.es (C.P.); ja.perez@umh.es (J.Á.P.-Á.); 2 Nutrition and Food Science Area, Preventive Medicine and Public Health, Food Science, Toxicology and Forensic Medicine Department, Faculty of Pharmacy, Universitat de València, 46100 Burjassot, València, Spain; manuel.salgado@uclm.es (M.S.-R.); noelia.pallares@uv.es (N.P.); emilia.ferrer@uv.es (E.F.)

**Keywords:** *Phoenix dactylifera*, dates, sugar, sweeteners, sugar reduction, alternative sweetener, sustainability

## Abstract

The current levels of added sugars in processed foods impact dental health and contribute to a range of chronic non-communicable diseases, such as overweight, obesity, metabolic syndrome, type 2 diabetes, and cardiovascular diseases. This review presents sugars and sweeteners used in food processing, the current possibility to replace added sugars, and highlights the benefits of using dates as a new natural, nutritious and healthy alternative to synthetic and non-nutritive sweeteners. In the context of environmental sustainability, palm groves afford a propitious habitat for a diverse array of animal species and assume a pivotal social role by contributing to the provisioning of sustenance and livelihoods for local communities. The available literature shows the date as an alternative to added sugars due to its composition in macro and micronutrients, especially in bioactive components (fiber, polyphenols and minerals). Therefore, dates are presented as a health promoter and a preventative for certain diseases with the consequent added value. The use of damaged or unmarketable dates, due to its limited shelf life, can reduce losses and improve the sustainability of date palm cultivation. This review shows the potential use dates, date by-products and second quality dates as sugar substitutes in the production of sweet and healthier foods, in line with broader sustainability objectives and circular economy principles.

## 1. Introduction

Presently, sugar is a main contributor to the onset of obesity and diabetes, which may be attributed to the elevated intake of added sugar in the processing of beverages, dairy products, desserts, cookies, candies, jams, among others [1,2]. The implications of excessive of added sugars in processed foods involve an excessive energy consumption, an impact on dental caries, and an increased prevalence of some chronic noncommunicable diseases (overweight and obesity, metabolic syndrome, type 2 diabetes and cardiovascular diseases) [3,4,5]. These disorders have manifested as significant public health challenges, prompting a call for the reduction of sugar consumption to enhance the nutritional profile of foods in alignment with public health recommendations [3,6,7]. The World Health Organization (WHO) promotes the preparation of guidelines to limit sugar consumption, defining the recommended threshold to be below 10% of the total energy intake in the diet and ideally less than 5% for optimal health benefits [6]. WHO’s sugar guidance adopts the concept of free sugars (FS), encompassing monosaccharides and disaccharides added to foods and beverages by manufacturers, cooks, or consumers, as well as natural sugars present in honey, syrups, fruit juices, and fruit juice concentrates [6]. The FS concept is considered more suitable than total or added sugars in this context.

The technological contribution of sugars must be duly considered in the strategies of sugar reduction or substitution due to key role of sugars in food processing, contributing to sensory quality, textural properties and shelf life. The modification or reduction of sugar content represents an important challenge for the food industry, potentially compromising the aforementioned functions [1].

Sugar, being a multifunctional ingredient, holds significant relevance in processed products. It imparts a sweet taste and mouthfeel in solid products and beverages, contributes to textural properties, participates in the Maillard reaction, resulting in brown crust color and appropriate aroma [1]. Additionally, sugar decreases water activity (Aw) in solid products, affects the freezing point, acts as a bulking and preserving agent, extends product shelf-life, and promotes lightness [8]. Among its roles, more relevant is conferring sweet taste to foods. Therefore, when sugar is substituted in a food product, maintaining the flavor, texture, and shelf life of the original product becomes necessary.

Sucrose is acknowledged as the reference sugar for sweetness and serves as a comparative standard for evaluating the sensory and technological attributes of potential alternative sweeteners [9,10]. Moreover, sucrose serves as a moisture retainer, thereby contributing to the extension of shelf life. Additionally, sucrose exerts a profound influence on the structure, appearance, and texture of numerous food, such as baked goods and chocolate, owing to its hygroscopic and crystallization properties [10,11].

The inherent hygroscopicity of sucrose plays a significant role in development the formation of a delicate texture, a heightened porous structure, and the expansion of baked products. Concurrently, the crystallization process of sucrose intricately participates in bestowing crispness and generating a crackling surface in biscuits and cookies [10]. Various researchers have documented the influence of sugar on the sensory and physical attributes of confectionery products, specifically cakes and cake-like items [12,13]. 

Moreover, sugar plays a pivotal role in binding moisture, and the moisture content varies across different sugar types. For instance, liquid sugars exhibit a higher moisture content compared to brown sugar, and brown sugar, in turn, contains more moisture than crystalline white sugar [14,15]. The impact of sugar in beverages is substantial, manifesting a multifaceted influence encompassing the provision of sweetness, flavor enhancement, enhanced palatability, increased viscosity, texture augmentation, and coloration. Simultaneously, sugar serves as a preservative by reducing water activity [16].

The physical attributes that sucrose possesses regulate fundamental processes that influence food texture, such as rheology, phase transitions of biopolymers and the distribution of water in the different phases of the food and texture-associated sensory attributes [17]. Their results indicated that the functional role of sugar, coupled with its functions as a plasticizer and humectant, significantly influences the rheology of biscuits. Consequently, these aspects can be strategically harnessed for the reformulation of biscuits to replicate the characteristics of their original counterparts [17].

Nevertheless, it is imperative to acknowledge that any reduction, elimination, or substitution of sucrose in food products may induce safety and quality-related undesirable effects [10]. Reducing the added sugar content of processed products to levels that do not compromise the properties and sensory characteristics of the final product poses a challenge for the food industry [1]. 

This review describes sugars and sweeteners used in food manufacturing. It considers the current potential for substitution of added sugars and highlights the advantages of incorporating dates as a natural, nutritious and sustainable alternative to artificial and non-nutritive sweeteners. This is in addition to the objective of promoting healthy eating behavior and the engagement of the agri-food industry to diversify our food systems towards sustainable production.

## 2. New Trends and Strategies for Sugar Reduction in Food and Beverages

Considering that processed foods constitute the primary source of FS intake, there exists substantial pressure on the food and beverage industry to engage in reformulation initiatives. The high demand to diminish added sugar content in processed products emerges as a principal concern for public health, necessitating governmental involvement through initiatives such as advertising regulation, regulatory policies, and taxation, among other measures [5]. Of notable concern is the report that the intake of added sugars in the diets of children and youth (aged 4 to 18 years) accounts for approximately 15% of their total dietary energy intake [18,19]. While sugars inherent in fruits and vegetables are enveloped within a natural tissue matrix, added-sugars and sugars found in syrups, honey, and fruit juices lack are free. Consequently, this last category is more readily available for digestive processes [5,18].

Several governments have instituted programs aimed at curbing sugar consumption. These initiatives encompass restrictions on the advertising of sugar-rich foods, salt, etc., the imposition of higher taxes on sugary beverages, and limitations on the availability of unhealthy products in vending machines [1,20]. 

One of the strategies employed to achieve sugar reduction in both solid and liquid food products involves the partial or complete substitution of sugar with a blend of diverse sweeteners. This approach aims to mitigate the risk of compromising the ultimate sensory characteristics of the products, striving to attain a taste and flavor profile comparable to that of sucrose [8]. Another initiative, in other countries, is the gradual reduction of sugar in liquid and solid foods, encouraging consumers to gradually adapt to the change in sweetness [21]. 

Beverages and food reformulation focused on reducing the sugar content of processed products is viewed as potentially beneficial in reducing sugar intake and improving health. These changes in the final product composition can compromise the final sensory characteristics in both solid and liquid foods, which implies the need for significant improvements in the sensory quality of sugar-reduced products [5,8,22]. 

Various methodologies for reducing sugar intake in food products center on alterations in food formulation or product re-design. These approaches comprehend direct sugar reduction, as well as the partial or complete substitution of sugar with sweeteners and bulking agents. Additionally, strategies involve cross-modal interactions or flavors with modifying properties (FMP), modifying the structure of sugar, and establishing a heterogeneous distribution of sugar within the food matrix [5,23].

The primary objective of these strategies is to effect sugar reduction while preserving the essential characteristics of the product, such as sweetness, color, and texture. Recent studies indicate that the simultaneous implementation of diverse sugar reduction strategies proves to be more efficacious than relying on a singular approach [16]. This underscores the complexity of reducing sugar content in food products and, at the same time, assuring consumer acceptance and satisfaction.

### 2.1. Changes in the Food Formulation

This strategy is intended to improve one or more properties of the product, including sensory attributes, safety, nutritional quality, among others [2]. The focus of this paper is specifically directed towards the reduction of added sugar to yield healthier products.

This strategy widely used in the development of low-sugar products involves the reduction of sugar content or the complete or partial substitution of sugar with low-caloric carbohydrates, non-nutritive sweeteners, or bulking agents. This approach is applicable to both solid foods and beverages, and is aligned with the target of improving the health profile of food products, particularly associated with excessive sugar consumption.

#### 2.1.1. Gradual Reduction of the Sugar Content in Foods

This reformulation strategy focused on gradually reducing the sugar content in food products, considering that consumers must accept the changes in sensory profile. Directly reducing the amount of sugar to lower the sweetness intensity of a product could affect product acceptance. In the case of beverages, direct sugar reduction could lead to a decrease in consumer palatability and acceptability, it has been studied that a gradual reduction is more effective than the stepwise reduction strategy because of its lesser impact on consumer sensory perception others [24,25]. Therefore, gradually reducing sugar in beverages can familiarize consumers to lower concentrations of sugar, reduce their preference for sweetness, and maintain consumer satisfaction with sugar-reduced products [26]. However, the impact of this strategy is limited, and the effect would be observed in the long term [16].

Several studies concluded that consumers did not perceive sugar reductions in the range of 6 to 11%, and it has even been shown that reductions of up to 20 to 30% do not seem to induce a major change in overall taste in both adults and children [26]. Reducing sugar in consecutive steps so that consumers do not notice any change in the sensory characteristics of the products is an effective strategy. The gradual reduction of sugar could promote the change of sweet preferences within a food category in favor of products with a lower sugar content [5].

#### 2.1.2. Partial or Total Replacement by Sweetener

This strategy is focused on the replacement of sugar by sweetener, nutritive sweeteners (NS) and non-nutritive sweeteners (NNS) depending on whether they contain calories, or natural and artificial sweeteners depending on origin. They maintain the sweetness of products without negative health effects (obesity, type II diabetes, and cardiovascular diseases) [16]. Nowadays, consumers can find a wide range of commercial processed products that contain at least one non-nutritive sweetener [5].

Since the 1980s, the use of non-nutritive sweeteners and low-calorie carbohydrates (prebiotic fibers) has been used to avoid the negative impacts (obesity, type II diabetes and cardiovascular disease) of high sugar intake, maintains the sweetness of products [27]. The discovery of sweeteners represented a key step for the innovation in the food technology (sweet products with non-caloric intake) [1]. To avoid undesirable effects of sugar reduction or elimination are needed to study the minimum sugar level and/or optimal sugar substitutes to maintain the basic functionality and final product quality.

NS, NNS, polyols, low-calorie carbohydrates (oligofructose, maltodextrin and polydextrose) and bulking agents are used to replace (partially or totally) the sucrose. The sugars commonly used in the food industry are: sucralose, maltitol, stevioside, sorbitol, isomalt, aspartame, erythrito, etc.) and bulking agents (unulin, maltodextrine, polydextrose, oligofructose, syrup, etc.), but they must be adapted to the food product, legislative standards and consumer preference [10]. The NS commonly used include: fructose, glucose, lactose and polyols. While NNS can include natural compounds (stevia, thaumatin and monk fruit), or synthetic compounds (saccharin, aspartame, sucralose, etc.), they are very sweet and tasty, but contain few calories [19]. 

Polyols (as erythritol, isomaltitol, lactitol, maltitol, sorbitol, mannitol, and xylitol), are food additives, and present insignificant caloric contribution, but a high sweetening capacity, being used in low quantities in food products [19]. In general, they are not cariogenic and do not cause glycemic response, thus being extensively used in hypocaloric diets, for diabetes patients and other specific cases where caloric intake must be controlled [28].

The inclination for a combination of non-nutritive sugar substitutes with sugar alcohols to produce a low-calorie bakery product has increased, with artificial sweeteners such as aspartame and sucralose providing sweetness and sugar alcohols providing the bulking properties.

In the last decades, the use of synthetic sweeteners (e.g., aspartame, sucralose, cyclamate, acesulfame and saccharin) has been commonin the industry to obtain low-calorie foods and beverages, due to their high sweetness, low cost, and calorie-free benefits [28]. 

In the case of beverages, the reduction of sucrose and its substitution with sweeteners, whether artificial or natural, can lead to a reduction in viscosity and have an adverse effect on the sensory and temporal profiles (manifesting as sensations like dry mouth and decreased viscosity) [16,23]. To avoid these undesirable effects associated with the incorporation of sweeteners into low-calorie beverages, carbohydrate gums and other food additives are introduced.

Sucralose is the most widely used sweetener in the beverage. The aspartame has been shown to exhibit stability in acidic liquid, and instability to heat and alkali; sucralose rapidly reaches peak sweetness and develops an undesirable residual sweetness [29].

New non-nutritive synthetic sweeteners in beverages have been reported such as neotame, advantame and alitame. Neotame has shown to be a good alternative to aspartame with a high sweetening power (7000 to 13,000 times sweeter than sucrose) and successful replacement has been reported up to 30% with no negative taste effects [16].

In the case of beverages, the sweetener aspects that should be considered to be selected are: specific solubility, pH stability, sweetness characteristics, processing temperature stability, temporal sensory profile and undesirable flavors [16]. Nevertheless, controversy exists about the safety over the use of artificial sweeteners in foods and beverages [5,15,30].

Artificial sweeteners share the same palatability as natural sugars, but the metabolic routes are different. The scientific literature shows artificial sweeteners are metabolized differently than natural sweeteners and may not all have the same metabolic impact, which can affect from the composition of the gut microbiota to the degree of digested and absorbed [30]. Artificial sweetener intake could affect body weight and glucose homeostasis through physiological mechanisms involving the gut microbiota, reward-system, adipogenesis, insulin secretory capacity, intestinal glucose absorption, and insulin resistance [30].

It is considered that reducing sugar through sweeteners and not sugar intensity does not reduce the intake of intensely sweet foods and, therefore, does not lead to the development of a preference for products with lower sweetness intensity. This approach has been associated with potential adverse health effects linked to the intake of sweeteners [31]. The activation of sweet receptors without the intake of sugar can lead to metabolic dysregulation [32]. This could be due to the decoupling of sweet taste from energy intake (learned relationship between sweet taste and post-ingestion responses), which may lead to the subsequent development of glucose intolerance. The metabolic response to carbohydrate intake depends on the relationship between energy intake and sweet taste [32]. 

Consideration of these impacts and the concern of consumers about artificial sweeteners is an important factor among other strategies than the use of artificial sweeteners as a means to reduce or replace sugar.

Natural sweeteners are widely used by the beverage industry because of the demand for clean label products. Stevia, a plant-derived sweetener, is present in some low-calorie drinks due to its good organoleptic properties, despite its bitterness, licorice taste and after-taste [16]. Bulking agents and flavorings can also be used in stevia beverage formulations to avoid these negative impacts on taste [33].

Another natural sweetener with future application is mogroside, a mixture of curcurbitane-type triterpenoid saponin, which is characterized by lower peak sweetness, a longer sweetness duration and after-taste (bitter, chemical and metallic taste) compared with sucrose. Sugar alcohols or polyols are good substitutes for sucrose, because they are minimally metabolized in the body, for example rythritol, calorie-free polyol, has been widely used in sugar-free carbonated beverages [33].

Finally, it is worth mentioning the other sugars (e.g., D-fructose and L-arabinose) and sweet proteins (miraculin, monellin, thaumatin, mabinlins, pentadin, curculin, and brazzein), may have wide potential application in the beverage industry as natural and clean sweeteners [33]. 

Natural sugars (unrefined sugar) are preferred because they have a high nutritional value due to their high concentration of healthy compounds (bioactive compounds, minerals, fibers, antioxidants, and phytochemicals), which balance the negative effects of refined sugar. Therefore, removing refined sugar or at least reducing its consumption should be promoted as a healthier option in food choices [34].

Natural sweetening agents (honey, xylitol, erythritol, maltose, maltodextrin, stevia, molasses, maple syrup, coconut sugar, agave nectar and date sugar) are presented as an important alternative to sugars, more attractive to consumers and a commercial opportunity for the food industry [34].

Natural sweeteners include the traditional sweeteners, natural sources of sugar. Traditional sweeteners are classed as NS and are obtained from bees (e.g., honey), plant and tree sap (e.g., maple syrup, agave nectar), fruits (e.g., carob syrup), seeds, roots (e.g., Yakon syrup) and leaves (e.g., stevia) and consumed within their natural matrix with minimal pre-processing. They are mainly composed of sugar of sucrose, fructose and glucose (at least 50% of plant-derived syrups and honey), small amounts of polyols, also contain additional nutritive compounds as proteins (< 1.4%), lipids (< 0.5%), dietary fiber (< 3%), and phytochemicals and small amounts of minerals (< 2%) and vitamins (< 0.02%), such as polyphenols [19]. Several positive impacts have been attributed to natural sweeteners, such as improving metabolic health, preventing weight gain and lowering blood glucose. These impacts could be because biomolecules with nutritional and health benefits (e.g., vitamins, phytohormones and minerals) present in NS appear to have the capacity to modify other physiological factors [27,34]. 

Some studies suggest the presence of phytochemical compounds in traditional sweeteners (honey and agave nectar) could contribute to a reduction in the glycaemic potency compared with glucose syrup and sucrose [19,27]. Phenolic compounds have a range of properties that could have a potential impact on nutrition and health (e.g., reducing the risk of cardiovascular disease, preventing neurodegenerative conditions and type 2 diabetes), as well as anti-oxidant properties. Therefore, by using fruits as a component for a sugar substitute, it will decrease the amount of daily sugar intake. Fruits have a sweet taste, and different studies have reported they are excellent sugar replacers. Ibrahim et al. [11] reformulated dark chocolate using palm sugar and dates as sugar replacers, which was well accepted by the sensory panelist.

Blending sweeteners can be a good alternative to avoid the negative impacts of certain sweeteners, obtaining a better result than any sweetener alone. A suitable combination of sweeteners can provide synergistic impacts on sweetness, stability and enhancement of flavor and temporary taste characteristics [35]. A suitable alternative blends NNS substitutes (providing sweetness) with sugar alcohols (providing bulking properties) to obtain a good low-calorie bakery product [15].

Sweetness enhancers, called positive allosteric modulators (PAMs), are compounds that have no sweet taste, however they increase sweetness intensity when used with a sweetener due to their synergist effect. According to DuBois and Prakash [36], PAMs are not able to activate the sweetener receptors, but their binding mode allows the sweetener to bind to the receptor. PAMs and sweetness are used together to reduce sugar due to their synergist effect and can potentiate the sweetness characteristic of sweetener. Furthermore, PAMs present the advantage that they do not cause bitterness, metallic taste or temporary sensory profile [25]. It has been reported that the combined use of PAM and nutritive sweetener allows for obtaining the same sweet taste using less sweetener, while the PAMs would be reducing off-flavors of non-nutritive sweeteners [37].

### 2.2. Flavors with Modifying Properties (FMPs)

This strategy presents a new type of flavorings, called flavorings with modifying properties (FMP) as an alternative to NNS. FMPs are able to activate responses in human taste receptors to increase sweetness, reduce saltiness and mask bitterness. Some FMPs interact with human sweet taste receptors and improve the sweetness perception [38].

FMPs have the property that they do not have a sweet or salty taste on their own but affect the taste or smell of other flavorings and food additives, and help maintain or improve the flavor profile of solid and liquid food. They facilitate the food industry to develop products more in line with regulatory requirements. It allows for the restriction of the use of sugars (e.g., sucrose, glucose, fructose), sweeteners (e.g., aspartame), and salt (sodium chloride) and masks some undesirable tastes such as the bitterness (e.g., potassium chloride), the persistent taste of certain sweeteners (e.g., stevia fractions), or soften the taste and astringency of vegetable proteins (e.g., soy, peas) [39].

### 2.3. Multisensory Interactions

Taste perception is a multi-modal sensory information integration process in the central nervous system that is dependent on temperature, medium, physical condition, age, and the information from other stimuli (smell, touch, hearing and sight) [16]. During the food intake, consumers used all their senses (multi-sensory integration) to identify the food product characteristics. Multisensory interactions are based on the role of food oral processing and microstructure and they are linked with sensory perception and texture [25,40], their understanding could be used to reformulate and develop healthier food products. 

Several studies have examined interactions between food-intrinsic and extrinsic factors in relation to sweetness to avoid a decrease in consumer satisfaction for the reduced-sugar products [25]. Nevertheless, it is difficult to determine the sweetness-enhancing multisensory factors as individual consumers have different responses to sugar-reduced foods and beverages [16,25].

This strategy focusing on multisensory interactions is based on the enhanced perception of sweetness intensity produced by aroma, color and other stimuli [23]. The use of multisensory interactions is another useful method for sugar reduction. Although the reduction of sugar content is limited, it has the advantage that sweeteners are not used. Multisensory integration can be studied from different perspectives: odor–taste, color–taste and sound– and tactile–taste interactions.

#### 2.3.1. Odor–Taste

Several studies on “sweetness enhancement” have reported volatile compounds that increased the sweet intensity and sweetness perception of foods, independently of glucose, fructose and sucrose content. It has been observed that the effect of odor on taste can be predicted from the sensory characteristics of the odor (e.g., degree of sweetness of an odor) [41]. In studies on different beverage and various odors, Bertelsen et al. [41] concluded that the sweetest odor “caramel” has been found to increase the sweetness of sucrose, while the odor with the least “sweet” odor suppresses the sweetness of sucrose. It should be noted that the effect of odor on taste can be predicted from the sensory characteristics of the odor, such as the degree of sweetness of an odor [42].

Furthermore, the enhancing effect on sweetness can be further increased as well as the optimization of the time phase-shift between odor and taste pulses [42]. When odorant (isoamyl acetate) and tastant (sucrose) pulses were presented out-of-phase, the sweetness intensity was enhanced by more than 35%, compared to a continuous sucrose reference of the same net sucrose concentration [42]. In addition, the sweetness-enhancing effect can be further increased by optimizing the time phase-shift between odor and taste perception [42]. When the odor and taste perception are presented the time phase-shift, the sweetness intensity increases by more than 35% compared to the same sucrose concentration [16,42].

Other authors concluded that the odor–taste interaction was an important strategy for sugar reduction in yogurt and preserving the desirable sensory characteristics [23].

It is known that the odor–taste interaction is produced via stimulating the olfactory receptors in the nasal cavity and the taste receptors in the tongue and mouth, and odor and taste stimulation activate cortical areas of the brain [42]. 

#### 2.3.2. Color–Taste Interactions

Color of food and beverages can affect the taste perception (e.g., sweetness threshold and intensity). Some authors showed the sucrose solutions containing red colorants obtained lower sweetness threshold [43], and red drinks were the sweetest, followed by blue and purple [44]. Different studies have shown that colors related with the natural ripening of fruits, such as red and yellow, are well suited to modulate the perception of sweetness, while colors opposite to the ripening of the fruit (e.g., green) may decrease sweetness perception; this can be due an association between specific colors and specific product attributes in the mind [45]. This could be due to the expectations that a color generates in the consumer, which affects the perception of sweetness [46,47]. Furthermore, other studies presented different results and reported that the impact of color on the perception of sweet taste was affected by the age of the population (adults or children) [47].

In addition, the package color of the beverages could, in the same way, affect the perception of sweetness, as it could involve the expectations of the product. More knowledge on this subject is necessary to confirm the impact of product or packing color on taste perception [16,28].

#### 2.3.3. Sound–Taste and Tactile–Taste Interactions 

Different studies have reported multisensory integration between taste and sound [16]. Although more knowledge is still needed on sound and touch–taste interactions in sweetness perception, it has been reported that beverages tend to be perceived as more enjoyable and sweeter when the beverages are presented in their own containers [48].

### 2.4. Sugar Structure Modification

Another strategy is focused on the reduction of the size of sugar particles to increase the surface/volume ratio per amount of sugar consumed, thus increasing the perception of sweet for the same amount of sugar when compared to larger sizes (20–100 μm). Using a spray dryer or nano spray dryer are techniques used to obtain these micro- and nanoparticles (350–500 nm). This approach presents some complications due to fact that the sucrose glass-transition temperature is close to 62 °C, making this material sticky [1]. An alternative to this strategy has been reported based on the addition of carriers (as inulin, pectin, lecithin, etc.) to the spray drying feed solution to increase the glass-transition temperature and avoid the stickiness problem. There are few studies on this subject [1,49].

The encapsulation of sweeteners has been used to enhance sweetener perception of alternative sweeteners. This technology allows the creation of structures or matrices with a controlled release of sweetness in food products and reduce the intense flavor of these alternatives, e.g., acesulfame-K, aspartame, thaumatin, xylitol, that need to improve their stability and sensory properties in food products [1].

### 2.5. Heterogenous Distribution

This strategy is based on the stimulation of taste receptors, liquid release, particle size and viscosity of foods to improve the sweetness in solid and liquid foods. The sugar content may also be reduced through the optimized stimulation of taste receptors to improve sweetness perception (heterogeneous distribution method), facilitated by liquid release from solid foods, the particle size and viscosity in solid and liquid food [1,23]. 

Richardson et al. [15] considered a new strategy of sugar reduction in confectionery-type products based on the sugar particle size, and they showed that different sugar particle size ranges (924–1877 µm, 627–1214 µm and 459–972 µm) have a significant impact on the physical and sensory properties. The studies concluded that small sugar particles (228 to 377 µm and 459–972 µm) showed an increase in the perceived intensity of sweetness in chocolate brownies, biscuits, so it could be used as a viable, economic and technological strategy to reduce sugar in baked products [14,15]. 

### 2.6. Encapsulation for Enhanced Sweet Perception

The encapsulation method is another novel strategy for the improvement of sweetener perception, based on the creation of structures that allow a controlled release of sweetness in food products and mask the intense flavor of these alternative compounds [1]. Different patents have been reported to encapsulate sweeteners and evaluate their effect to improve their application [50]. Alternative sweeteners such as acesulfame-k, aspartame, thaumatin and xylitol are suitable for use in the encapsulation strategy, as this methodology allows for the improvement in their stability and sensory properties in food products [51,52].

## 3. Nutritive, Non-Nutritive and Traditional Sweeteners

As mentioned, the high intake of sugar not only relates to a high accumulation of body fat, but also can concurrently increase the risk of other adverse health conditions, such as type 2 diabetes or cardiovascular diseases. With this regard, and considering the demand of the population for healthier and more natural foods, over the past several decades the food sector has been focused on sugar substitution in different foodstuffs, thus replacing the traditional sweeteners like glucose or sucrose. Overall, healthier products would be obtained, with the desired health benefits to consumers, and satisfying at the same time their demand for sweetness [53].

In line with the above, artificial, non-nutritive sources are nowadays the most common alternatives [54,55,56]. However, despite being considered as calorie-free sources, they can sometimes involve metabolic diseases, such as type 2 diabetes or cardiovascular diseases as well [57,58]. For that reason, there is a continuous need for natural alternatives which can be extracted from natural sources [27,53]. Therefore, the classification for sweeteners can be organized considering both the origin of these healthy products (synthetic, natural), or their nutritive power (calorie-load or calorie-free) [59]. The main sweeteners and specific substances employed worldwide are detailed below.

### 3.1. Synthetic Sweeteners

Artificial sweeteners or sugar substitutes are synthetic substances employed to replace sugar during the sweetening process of several products, such as sweets, preserves, dairy products and beverages. The molecules in artificial sweeteners include principally sulfa, dipeptide and sucrose derivatives. These compounds provide a sweet taste without increasing caloric intake and blood sugar levels. Therefore, due to their high efficiency (30–13,000 times the sweetening power of sucrose), a small amount of these compounds provides high sweetness without a caloric intake increase [60,61]. Nonetheless, the amount of use must be safe to guarantee consumers’ health [60]. Some of them including aspartame, neotame, saccharin, acesulfame-k, sucralose and advantame, which have been approved as food additives by the Food and Drug Administration (FDA) [27,59]. 

Synthetic sugar substitutes must have a sucrose-like taste quality and demonstrate safety, with non-toxicity and cariogenic capacity and no effects on blood glucose or insulin. However, some studies suggest that these non-caloric sweeteners might cause an ambiguous psychobiological signal that confuses the body’s regulatory mechanisms [61]. 

Nowadays, the major part of sweeteners available on the market are synthetic compounds. However, they must grant legislative approval and the regulatory requirements of each country. These compounds have undergone a wide safety evaluation process by international and national regulatory food safety authorities, such as the FAO/WHO Joint Expert Committee on Food Additives (JECFA), the US Food and Drug Administration (FDA) or the European Food Safety Authority (EFSA) [62]. Moreover, these authorities continuously review and evaluate any new safety information related to them. In this sense, some problems have been attributed to some of these compounds in terms of their stability, cost, quality of taste and safety [61]. However, over the past few years, because of health concerns, consumers are demanding more natural and healthy foods that are produced in a sustainable way. Thus, food manufacturers are looking for natural and functional sweeteners to be applied in foods [60].

#### 3.1.1. Aspartame

Aspartame (E-951) was discovered in 1965 and was the first sweetener approved by the FDA. Its flavor characteristics are acceptable. This artificial sweetener reduces food intake and may assist with weight control. Some studies evidence that their consumption does not influence blood pressure, glucose and lipid profiles, being a safe option for type 2 diabetics. However, its consumption is still controversial since some studies have associated it with adverse health effects, such as interference of neuronal cell function, hepatotoxicity, kidney disfunction and oxidative stress in blood cells. Moreover, its usage is not recommended for people who suffer from phenylketonuria, since they cannot metabolize phenylalanine, a compound involved in the aspartame synthesis [27,63]. To ensure its safety, the European Commission (EC) has established an acceptable daily intake (ADI) of 40 mg/kg bw/day [64].

#### 3.1.2. Neotame and Advantame

Neotame (E-961) and advantame (E-969) constitute derivatives of aspartame. Neotame is an isomer of aspartame, while advantame is an N-substituted derivative of aspartame and vanillin. Both artificial sweeteners are sweeter than aspartame, and show approximately 13,000 and 20,000 times the sweetening power of common sugar, respectively. Neotame was approved by the FDA in 2002, while advantame was approved in 2014. Similar to aspartame, neotame and advantame present no adverse effects on the human metabolism, constituting a safe option for type 2 diabetes patients [27].

#### 3.1.3. Sucralose

Sucralose (E-955) is also a non-caloric sweetener, that does not break down in the body. Its sweetening potential is approximately 600 times higher than sugar. It constitutes a good option for many industrial applications due its stability at different pH conditions and temperatures. Some studies suggested that this artificial sweetener could interfere with digestive processes, increasing glucose and insulin levels in the body and resulting in weight gain and diabetes risk. Nonetheless, studies related with absorption, distribution, metabolism and excretion pointed out that sucralose is mainly eliminated through fecal excretion, without being absorbed or digested in the organism [27]. The EU Scientific Committee on Food (SCF) established an ADI of 15 mg/kg bw/day) [65].

#### 3.1.4. Saccharin

Saccharin (E-954) was discovered in 1878 and is the oldest and most studied of all sweeteners. This compound presents good technological characteristics, such as stability at low pH and high temperatures. Moreover, it is not metabolized in the gastrointestinal tract and does not alter insulin levels [27,63]. 

This compound has been involved in some human health concerns, since the USA FDA considered its prohibition as a consequence of some studies that related it with bladder cancer in rats. However, in subsequent studies the relationship between saccharin and bladder cancer has not been demonstrated in humans. The EC has established an ADI of 5 mg/kg bw/day [66]. 

#### 3.1.5. Acesulfame-K

Acesulfame-K (E-950) was discovered in 1967 and is one of the most common low calorie artificial sweeteners. It constitutes a thermostable component, with good properties to be manipulated. This compound presents around 120 times higher sweetening potential than sugar although it bears a bitter taste, therefore it is usually employed in combination with other sweeteners. It cannot be metabolized without increasing the caloric intake [67].

Acetoacetamide, a breakdown product of acesulfame-K, might be toxic for humans at high concentrations and its consumption has been related by some studies with genotoxicity and the inhibition of glucose fermentation by intestinal bacteria. However, mores studies including bioassays are necessary to clarify this issue [27,63]. The ADI established by EC for acesulfame-K is 9 mg/kg bw/day [67].

Nowadays, there is a need to find healthier substitutes to refined sugar. In this sense, an ideal alternative sweetener should a guarantee lower calorie content and helps to prevent dental decay and diabetes. Moreover, it should present some characteristics to be incorporated into the food during its manufacturing, such as water-solubility, stability in acidic and basic pH, and being metabolized in a wide range of temperatures. It also should demonstrate its non-toxicity [53]. 

### 3.2. Nutritive Natural Substitutes

In regard to the above, natural products are normally appealing to the population since they attribute their consumption to health benefits. Thus, as previously mentioned, the production of natural substances by the food sector becomes a valuable hotspot. Concretely, it is well-known that nutritive natural sweeteners involve low glycemic potency and fructose content. In addition, a wide range of bioactive compounds, such as polyphenols or vitamins, can be found in these sources, as well as others as minerals or phytohormones. Overall, these substances are considered as safe, and also bring nutrition to the human body, with positive effects in terms of improving metabolic health, preventing weight gain or lowering blood glucose [19]. 

Many reports in the literature have already described the main natural, nutritive substances for sweetening [27,53,68]. Among them, honey, molasses (viscous substances come from the refining process of sugar cane), maple syrup, agave nectar or coconut sugar are worthy of note. Briefly, the main properties, composition, and applications of these substances are summarized in Table 1.

Concurrently, the power of polyols or some kind of oligosaccharides should be also mentioned in this section. The former are commonly used in the food sector since they also entail a distinguished sweetness (50–100% sucrose [19]), and are naturally present in fruits, vegetables, or natural fermented foods. The most common are xylitol, mannitol, sorbitol, or erythritol, all of them with reduced calorie power [69]. Further, some specific oligosaccharides are gaining attention. In this context, 2′-fucosyllactose (2′-FL) or trehalose (TRH) are noticeable, since they can bring both energy and potential benefits, for instance, improved immunity, blood sugar regulation and weight loss [59]. Trehalose (2′-FL) is commonly found in milk, whereas TRH is widely present in plants, bacteria, or fungi. They have been authorized as sweeteners and additives for commercial issues.

### 3.3. Non-Nutritive, Natural Sweeteners

Despite the positive properties of the above natural sweeteners, most of them involve a high-calorie content. Regarding that, and also the population concerned about overweight, diabetes, and their health-associated concerns, the development of alternative natural-calorie-free sources has become crucial. In this context, steviol diterpene glycosides recovered from Stevia Rebaudiana are worthy of mention. These substances normally exhibit a high sweetness power, concretely between 40 and 450 times stronger than sucrose, and represent a proportion ranging from 4 to 20% of the dry stevia leaves. In addition, they showed valuable health benefits, as reported [27,59]. Concurrently, the presence of biologically active compounds, such as polyphenols, should be noted in stevia [70]. Among them, flavanols, flavones and tannins are remarkable, as well as hydroxybenzoic and hydroxycinnamic acids. Therefore, in addition to its low-caloric content and the high sweetness intensity by the glycosides structures, Stevia Rebaudiana also entail pharmaceutical and medicinal applications, for instance, anti-cancer, antioxidative, or anti-inflammatory effects [71], possibly making them the most potential natural sweetener.

Briefly, the most valuable steviol glycosides are stevioside and rebaudioside A [70,71], with beneficial health effects, as mentioned. Additionally, others such as rebaudiosides B, D and M should be mentioned [59,71].

Finally, apart from these steviol derivatives, glycyrrhizin is considered another glycoside potential sweetener [59]. This pentacyclic triterpenoid is a recognized bioactive ingredient of licorice, and exhibits around 170-fold higher sweetness compared to sucrose. It presents a wide pharmacological activity such as anti-inflammatory, antitumor or hepatoprotective agent, among others [72].

### 3.4. New Natural, Healthy Alternatives to Sweeten: Date Fruit

The soluble date sugar extracted from date fruit would be a suitable alternative to refined sugar, with a lower glycemic index than sucrose. 

The composition of date fruits rich in carbohydrates (70–80%), most of them in the form of sucrose, fructose and glucose, and in other phytochemicals make them an ideal source for the production of natural sugar. Furthermore, date fruits contain a good amount of dietary fiber ranging from 6.5 to 11.5% (of which 6–16% is soluble), which can help to meet the requirements of a balanced diet [73,74]. 

Date fruits also show beneficial properties, such as antitumor, anticancer, antioxidant, anti-mutagenic, anti-inflammatory, gastroprotective, hepatoprotective and nephroprotective effects [75,76,77]. Moreover, date fruits have demonstrated antibacterial activity attributed to bioactive compounds like phenolic molecules [78].

Date syrup constitutes the principal derived date product, and its usage is contemplated as one of the oldest practices in the production of sweeteners. It is employed in the food industry to be incorporated to foodstuffs such as jams, marmalades, concentrated beverages, chocolates, ice cream, confectioneries, and honey. The syrups obtained from date palm present high amounts of sugars, minerals (potassium, iron, magnesium and calcium), vitamins (B1 thiamine, B2 riboflavin, nicotinic acid, A and C) and a distinguished antioxidant activity, mainly related to their high content in phenolic compounds. Moreover, date syrup is rich in unsaturated fatty acids (such as oleic, linoleic, palmitoleic and linolenic acids) [27].

Currently, liquid date sugar is obtained from date fruits by ultrasound-assisted extraction (temperature of 60 °C, extraction time of 30 min, and liquid to solid ratio of 7.6 mL/ga and L/S ratio) [74]. However, some authors proposed alternatives to conventional extraction such as enzymes (pectinase and cellulase) and ultrasonically-assisted methods to extract syrup date, with high efficiency at shorter extraction times [79,80].

## 4. Sustainability and Valorization of Date Palm

**Figure 1:**
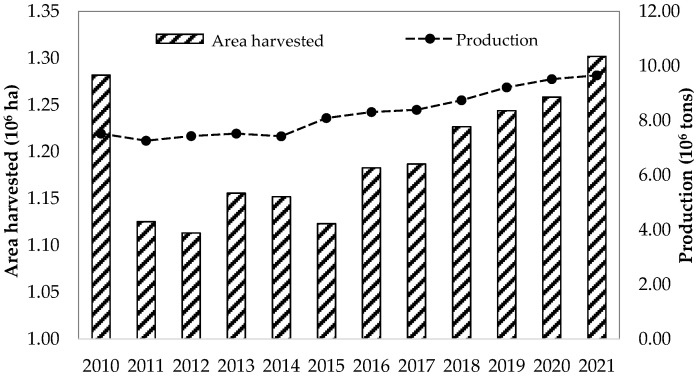
World evolution of the date palm area harvested and the date production in the period 2010–2021 [82].

**Figure 2:**
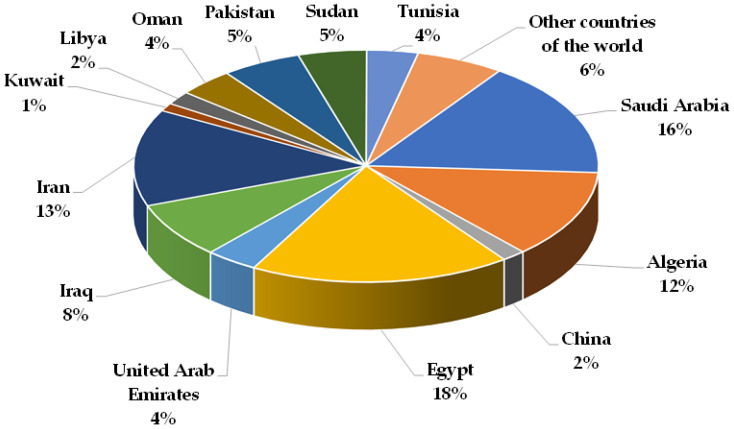
World distribution of date production in 2021 [82].

Date palm (Arecaceae family; *Phoenix* genus and *P. dactylifera* species) is considered one of the most ancient cultivated trees in the world and is mainly cultivated in arid and semi-arid areas of southern Europe, North African and southern Central Asian countries [81]. Date palm cultivation has increased over the last decade (Figure 1) [82], with world date production of about 9.7 × 106 tonnes in 2021, where Egypt, Saudi Arabia, Iran and Algeria are the main producers (Figure 2) [81]. In the European Union, the largest palm groves are in Spain, located in Elche and Orihuela (Alicante province, southeast of Spain). Both palm groves are protected spaces because they are considered unique cultural and historical landscapes of great value [83,84].

The date palm excels in challenging environments, thriving in dry climates with low rainfall, high evapotranspiration, and salinity tolerance. It withstands temperatures from 18 °C to 50 °C and short frost periods as low as −5 °C [85,86]. High humidity promotes phytopathogen proliferation, inflorescence rotting, and the production of soft, sticky fruits [81,86]. Hot and dry winds reduce receptivity, and strong winds can disperse pollen, break fruit stalks, and damage developing fruits [81,87]. Date palms thrive in sandy to sandy-loam soils [81,88]. They are highly tolerant to salinity (up to 12 dS/m electrical conductivity), but their production decline starts at 4 dS/m [82,89]. Excessive soil salts result from scarce rainfall and the overexploitation of saline aquifers for irrigation [90]. 

Climate change threatens food security, necessitating diversified food systems. Promoting climate-resistant foods such as the date palm aligns with the UN’s Zero Hunger goal, as the date palm seems less affected by climate change [86,91]. Palm groves combat desertification, create a microclimate, preserving agrobiodiversity and support various animal species, benefiting local communities [92,93,94,95]. Intensive date palm cultivation brings environmental challenges, e.g., leading to soil salinization [96,97,98,99,100,101] or reduced livestock presence [102].

### Date Uses and Valorization of By-Products of the Date Palm

Date is a fruit with high nutritional value and is very healthy, its main constituents being carbohydrates and dietary fiber, as well as minerals (especially potassium), vitamins, antioxidant phenolic compounds and carotenoids and to a lesser extent, it also contains proteins and lipids [103]. For this reason, the main use of date is its fresh consumption, especially the highest quality dates (first- and second-grade dates). Lower quality dates are classified as third-grade and cull date. The former is processed and cull dates are destined for animal feed [104]. The following products are obtained from the processed dates:

− Date syrup: this date by-product is obtained by hot aqueous extraction (60 °C) of the date juice and subsequent vacuum evaporation of the extract obtained. Date syrup is used as an ingredient in the preparation of bakery products, ice creams, jams, beverages, etc. [105]. This product has also been used as a sweetener to replace sugar in the preparation of different desserts [106,107,108] and non-alcoholic beer [109]. In addition, it is added to prebiotic milk and yogurt to improve its organoleptic properties [110,111]. Date syrup can also be used as a carbon source for bacteria in various fermentation processes where the following products are obtained: alcohol, date wine, antibiotics, organic acids, bakery yeast and unicellular proteins [104].
− Date paste: this product is obtained from the grinding of ripe pitted and skinless dates, which have been cooked in hot water or steamed [104]. Date paste has been added to meat products to improve their textural properties, reduce their fat content and increase their concentration of dietary fiber [112], as well as to reduce the oxidation of pigments and lipids during storage of pork liver pâté [113]. This by-product is also used to prepare jam and candies due to its high sugar content [105].
− Date pit: this part of the date fruit can be used for animal feed or added in powder form to different foods to increase its dietary fiber content [105]. Also, the date pit can be treated by pyrolysis to obtain bio-oil and biochar [104]. This biochar has shown very good properties for the removal of organic and inorganic contaminants present in wastewater and drinking water [105]. In addition, oil can be extracted from the date pit with different uses in the food industry, such as cooking or frying oil and for the preparation of margarines and mayonnaises, and the presence of a wide variety of phytochemicals in this oil means that it is also used for the formulation of cosmetic and pharmaceutical products [114]. Date pit oil has also been employed for the production of bio-diesel [115] and as feedstock for the production of polyhydroxyalkanoates with use in the synthesis of biodegradable plastics [116].

On the other hand, the fresh date and methanolic and aqueous extracts of date have been traditionally used for medicinal purposes for the treatment and prevention of different diseases [117].

Also, the cultivation of the date palm generates large amounts of agricultural residues from the pruning operations of the leaves with signs of senescence and the bunches with the harvested dates. These residues can be used to make paper, produce particleboard composites, obtain energy (through thermal treatment by pyrolysis and combustion or through anaerobic digestion) and manufacture composites of natural fiber for use in the automotive industry [85,118]. Likewise, date palm biomass residues have been co-composted with residues from different origins to produce biofertilizers compost [119].

## 5. Nutritional and Functional Properties of Dates

The diversity of date palm cultivars offers various choices, and their adaptability to different climates contributes to their global popularity. The escalating interest in date fruits and their derivatives is attributed to their role as a highly nutritious and plentiful fruit, and as a cost-effective source of numerous macro- and micronutrients, such as minerals, vitamins, antioxidants, and dietary fibers as well as secondary metabolites essential for human health. The carbohydrates, primarily sugars, constitute the majority of date fruit composition [103,120,121,122].

Date fruits consist of two main parts: the edible flesh (pulp), representing 85–95% of the total weight, and the seeds (or pits), comprising 5–15% and serving as a notable byproduct in date palm processing. The nutritional composition of date pits, rich in protein, fat, and dietary fiber, has sparked interest in novel functional food applications [122].

With an energy value ranging from 300 to 350 kcal/100 g, date fruits exhibit varying carbohydrate compositions influenced by cultivar types and ripening stages. Date pulps contain easily digestible sugars, primarily glucose, fructose, mannose, maltose, and sucrose constituting over 80% of dry matter [123]. The sugar composition varies, with sucrose predominant in dry dates, while soft dates are characterized by glucose and fructose. Additionally, the dietary fiber content in date pulp varies widely, including insoluble cellulose, hemicelluloses, pectin, hydrocolloids, and lignin [103,120,121,122,124,125].

In addition to carbohydrates, dates emerge as an exceptional source of proteins with a protein proportion of 2.5–6.5 g/100 g, fats, dietary fibers, and a spectrum of essential minerals and vitamins (rich in B-vitamins) [124]. Dates contain more than twenty different amino acids, which is uncommon in fruits [123].

Dates are established as a superior dietary fiber source compared to cereals. Additionally, dates contain health-promoting β-glucan, that shows potential anticancer properties [123]. Date seeds, with higher dietary fiber content than date flesh, present an opportunity for excellent sources of dietary fiber in food processing [122]. Protein content in date pulp ranges from 1.2% to 6.5%, while date seeds contain 5.1–7% protein, including essential amino acids such as glutamic acid, aspartic acid, and arginine [122]. Dates exhibit low fat content, mainly concentrated in the skin. Date pits, on the other hand, have a significantly higher oil content, making them a potential source of edible oil rich in unsaturated fatty acids [121,122,126].

Noteworthy nutrients include potassium, vital for a healthy nervous system and overall balance, phosphorus collaborating with calcium for bone strength and growth, magnesium, copper, zinc and selenium crucial for cell growth and repair, and iron essential for red blood cell production, facilitating nutrient transport to cells throughout the body, are also found in dates. The low sodium content in dates aligns with recommended daily intake levels [103,120]. Date seeds also contain various dietary minerals, further enhancing their nutritional profile [121,122,123,125]. 

Date pulp and seeds are rich in biologically active molecules, with variations based on the cultivar of origin [127], specifically polyphenols, mainly flavonoids [128], carotenoids and phytosterols [121], highlighting their nutritional quality. These phytochemical compounds underscore the antioxidant, anti-diabetic, anti-obesity [129], hepatoprotective [130] and neuroprotective actions [131] and anti-lipidemic properties of date fruits, contributing to their overall health benefits in human consumption [103,120,121,124,132,133]. The study carried out by Alsukaibi et al., [134] indicated the presence of various components in date fruits, responsible for cytotoxicity against cancer cells. Dominant phenolic compounds, such as q-coumaric, ferulic, and vanillic acids, were identified. Antimicrobial assays demonstrated notable biological activities, for second-grade dates. Significantly, these extracts displayed extensive antimicrobial activity against various pathogens [103,120,123]. Alsukaibi et al., [134] found that date kernel (seed) is a natural source of polyphenols that have potential antibacterial activity.

Polyphenolic compounds, particularly phenolic acids and flavonoids, represent primary secondary metabolites in plants. Date fruits emerge as a noteworthy source of these compounds, surpassing other fruits, and can be found in both the pulp and seeds. The concentration and diversity of these phytochemicals generally prevail in the pulp compared to the seeds. The concentration of polyphenolic compounds is contingent upon factors like cultivar, ripening stage, and environmental conditions. Analyses of phenolic acids in date fruit pulp from various studies reveal variations in composition and concentration, with gallic acid frequently standing out [122].

In parallel, date fruit seeds contribute to the pool of phenolic acid compounds [114,135]. Notably, gallic acid and syringic acid are major compounds in date seed extracts from different cultivars. The concentration of these compounds varies among cultivars. Additionally, studies on date fruit seeds from various regions report the presence of phenolic compounds like ferulic acid, vanillic acid, and p-coumaric acid, showcasing the diversity in phenolic acid composition [122]. 

Regarding flavonoid content, analyses reveal quercetin as a primary component in date fruit pulp, while date seeds exhibit flavonoids such as rutin, quercetin, and luteolin. The predominant flavonoids in date seeds include catechin, epicatechin, quercetin, and quercetin hexoxide. Rutin is identified as a major flavonoid in date seeds from specific cultivars. Overall, it is crucial to note that the concentration of flavonoids in date seeds tends to be lower than in the pulp [122]. 

The findings propose that second-grade dates hold substantial promise as efficient, safe, and cost-effective natural antioxidant compounds. This potential creates new possibilities for their utilization in the functional food and nutraceutical industries, highlighting the diverse benefits of dates beyond their nutritional content [103,120].

## 6. Food Applications of Date as a Sweetener

Elevated sugar consumption has been associated with negative health effects, including dental caries, type 2 diabetes, and cardiovascular diseases, particularly among the demographic of children and adolescents [136,137]. A noteworthy proportion of current consumers is actively seeking healthier alternatives within their lifestyle, emphasizing a change toward a more health-conscious diet. This includes an effort to reduce sugar intake and substitute refined sugar with naturally sourced sugars. Consequently, there exists a considerable interest in the development of food products incorporating natural and healthier sugars or sweeteners derived from natural sources [27]. Research has demonstrated that alternatives to sugars from natural sources, as is the case of the date palm fruit, contains significant levels of bioactive compounds, such as antioxidants, minerals, fibers, and other phytochemicals. 

Due to the healthy and medicinal properties associated with the consumption of dates and its products, based on the nutritional and bioactive composition (rich in dietary fiber, minerals, carotenoids, vitamins and phenolic compounds), this fruit is desirable to incorporate into the diet [123,138,139,140]. Furthermore, this fruit can be regarded as an emerging and potential candidate as alternative for substituting refined sugar in the processing of solid, semi-solid, and liquid food products [138,141]. It should be noted that food matrices are an optimal carrier to facilitate the availability of the biomolecules present in date fruit [139]. This approach not only enhances health benefits and adds value but also contributes to the revalorization of date products and by-products, in that way promoting circular economy principles within the food industry [142]. 

Dates, ready-to-eat date products, and date-derived products such as syrup, juices, spreads, paste, and liquid sugar [143] possess the potential to function as sweeteners while providing essential vitamins, minerals, phytochemicals, antioxidants, and other health-promoting compounds. These properties contrast with those of refined sugars, which are characterized by empty calories. Additionally, dates exhibit versatile applications beyond their role as sweetening agents, extending to functions as coloring and flavoring agents [144]. Consequently, dates may be utilized as ingredients in specific foods in which sugar is a fundamental component by providing sweet taste and functional properties. Such applications involve beverages, confectionery, desserts, baked goods and dairy products, as shown in Table 2.

A large number of studies focus on the addition of date and date products (syrup, extract and powder) to dairy products [143,144,145,146,147], dessert [107,148] and beverages [109,149,150], candy [151], biscuits [152,153,154,155,156,157,158,159], bread [160,161,162], snack bars [163,164,165] and flakes [166]. This integration represents a viable and effective strategy for the creation of novel functional foods, with an improvement in functional and nutritional properties, and good sensory attributes [142].

Amerinasab et al. [143] incorporated varying concentrations (1 to 9%) of date liquid sugar as a substitute for added sugar in the production of dairy products. Their conclusions indicated that yoghurts containing 6% exhibited optimal pH, total titratable acidity, and color characteristics. These yoghurts also demonstrated elevated firmness and viscosity, reduced syneresis, and received the highest scores in sensory evaluations for texture, aroma, flavor, and overall acceptability. Furthermore, a discernible enhancement in antioxidant activity and phenolic content was observed in these yoghurts.

Abdollahzadeh et al. [146] enhanced the nutritional composition of date-flavored probiotic fermented milk by supplementing it with various combinations of date extract at concentrations of 4%, 8%, and 12%. The study revealed a proportional increase in antioxidant activity. Moreover, the probiotic content, specifically Lactobacillus acidophilus, consistently exceeded 6 log10 units throughout the product’s shelf life. The authors concluded that date extract presents itself as a viable candidate for enhancing the nutritional profile of probiotic dairy products.

Other authors have added date syrup as a natural and nutritional additive in yogurt [144,145,147], a fermented milk beverage to [149], to produce healthy and nutritious flavored milk beverage with lower amounts of added sugar thus improving its nutritional properties. Djaoud et al. [107] concluded that the incorporation of date by-products (syrup or/and power date) as substitute sugar, could be an alternative to formulate new dairy dessert. They showed dairy dessert with syrup date exhibited the highest total phenolic content, DPPH inhibition, and reducing power, followed by mixed dairy dessert.

Dates have been studied as natural sweeteners for sugar replacement in chocolate products [11,142,167]. These authors replaced sugar by date syrup or powder, alone or with other sweeteners, as an alternative sweetener in the production of chocolate products, improving the taste and flavor and the healthy and physicochemical properties [11,142,167]. Prebiotic chocolate milk (non-fermentative dairy product) with a high sugar content has been reformulated using date syrup as a natural sweetener and inulin as a prebiotic, resulting in an optimal prebiotic chocolate milk, with the added value of having a natural and cost effective as sugar replacer [110]. Additionally, date seed could be used as a good healthy alternative for cocoa powder in chocolate processing, showing that the chocolate sample manufactured with 4% date seed powder was significantly superior in the degree of taste, aroma, and texture and in bioactive compounds (fiber and phenol content) [168].

Other studies have been directed towards reformulation strategies aimed at reducing or replacing sugar content in low-moisture baked products such as biscuits and bread. Aljutaily et al. [158] demonstrated that biscuits supplemented with 5%, 10%, and 15% date fiber exhibited functional anti-obesity properties in obese albino rats. This suggests a potential biological impact of date palm fruit on body weight control in this particular animal group. It has been presented that date syrup exhibited similar effects to sucrose on thermal properties [152], and this aspect can be potential for optimizing sugar replacement in biscuits and dough by utilizing date syrup and liquid sugar [152,156]. 

Other studies have focused on the incorporation of date powder and flour [153,154,157,159] in biscuit production, resulting in enhanced nutritional value. However, there is a limitation, with a recommended replacement threshold of 10–20% to avoid adverse effects on sensory analysis and physical characteristics. In addition, other varieties of palm, such as *P. canariensis* [155] have been studied in biscuit production in order to develop a new food application for these fruits. These authors evaluated the addition of date powders as a replacement to wheat flour or sugar, and obtained novel biscuits with higher fiber and polyphenolic content [155].

Several studies have researched the effects of substituting sugar with date products in bread production, with the aim of enhancing its nutritional value [160,161,162]. These studies have demonstrated that the inclusion of date flour and paste can approach the functionalities of sugar in bread production, contributing to improvements in crust color and flavor. Furthermore, this substitution leads to enhancements in the nutritional profile of the bread, characterized by increased levels of protein, minerals, and fiber. These nutritive improvements are attributed to the supply of bioactive compounds and dietary fiber from dates, with minimal baking losses [162].

Dates and their products have shown a great future commercial opportunity in snacks and fruits bars with improved nutritional value and functional properties with the increase in the date content [162,163,164,165,169,170]. The conventional snack bars generally include natural sweeteners such as honey and dried fruits, but they can be replaced by other natural substitutes by date and date products which present optimal technological qualities and lower price [165]. Different studies have shown the potential application of dates, rich in functional and bioactive ingredients such as phenolics and flavonoids, to develop balanced, nutritious, and functional date-based bars [170].

**Table 2:** A selection of studies on the use of date and date products as sweeteners in food processing and its main results.

Food	Way of Incorporation	Concentration Used	Main Results	References
Fruit yogurts	Date liquid sugar (DLS)	1–9%	Higher phenolic compounds and antioxidant activity Yogurts with 6% DLS had the highest scores	[143]
Flavoring yoghurt	Date syrup	6.0, 8.0 and 10%	Higher acidity, solids, proteins and ash Decreased fat, pH, total bacterial count and increased lactobacilli count The best level of addition was 8%	[144]
Flavored drinking yogurt	Date syrup	5 and 10%	Higher acidity Increased viscosity Sensory characteristics acceptable	[145]
Probiotic fermented milk	Date extract	4, 8 and 12%	Higher antioxidant activity and acidity Count reduction Lower pH and syneresis No negative sensory impact	[146]
Functional yoghurt	Date syrup	5%	Higher the nutritional value Enhanced the quality and overall acceptability	[147].
Fermented milk beverages	Date palm with camels’ milk and goats’	10%, 20% and 30%	Improved the composition, viscosity, microbiological quality and acceptable sensory attributes Higher acceptable sensory at 10% and 20%	[149]
Dairy Desserts	Date syrup (DS) and dried date powder (DP)	16% with the rates: DP/DS = 2; DP/DS = 1 and DP/DS = 0.5)	Enhanced the final product texture. Improved antioxidant activities	[148]
Dairy dessert	Date syrup (DS) date powder (DP)	14% DS and 2% DP	Enhanced the dry matter, lipids, proteins, total phenolic, and antioxidant activity	[107]
Dark chocolate	Dates syrup (70° Brix)	25%	Better physicochemical Well accepted sensory	[11]
Chocolate spread	Date seed powder	2, 4, 10%	Increased crude fiber, total phenol, antioxidant activity and value of L*and h Better in 10% of date seed Decrease in the a*, b* and C	[169]
Chocolate	Date powder	17.94, 19.86 and 25.16%	Improved the taste and flavor of the product	[167]
Prebiotic chocolate milk	Date syrup	4 and 10%	Increased the total solids 10% of date syrup was selected as the optimum	[110]
Biscuits	Date syrup	10, 20, 30, 40, 50, and 60%	Decrease hardness. Lower fracturability Darker cookies	[152]
Biscuits	Date power	5, 10, 20 and 40%.	Increased carbohydrates, crude fibers, ash, crude fat, moisture and protein Decreased physical characteristics of cookies The best was at substitution of 10%	[153].
Biscuits	Date palm flours	15, 17.5, 20, 22.5, 25, 30%	Higher in crispiness Lower the spread ratio Increased fiber content	[154]
Biscuits	Date powder from *P. canariensis*	5%, 7%, 9%, and 11%	Increased in hardness, polyphenol and fiber content, and antioxidant activity The maximum acceptable was 9% and 7%, Two-fold fiber and four-fold polyphenolic content	[155]
Biscuits and Dough	Date syrup and date liquid sugar	Sucrose was replaced at 0, 20, 40, 60, 80 and 100%	Increased pH, cohesiveness and decreased softness and adhesiveness in dough Lower pH and higher ash, moisture, density, antioxidant, mineral content texture and darker color in biscuits,	[156]
Biscuits	Date powders	20 and 30%	Increased in moisture content, starch, ash and fiber content 20% the best in sensory quality	[157]
Biscuits	Date fiber	5, 10 and 15%	Significant positive effects Functional anti-obesity properties resulting in body weight. Lower levels of glucose, and cholesterol in rats	[158]
Biscuits	Date powder (+chickpea)	10, 20, 30 and 40%	Higher ash, far, fiber, fat and protein Lower carbohydrate Higher spread factor and spread ratio Decreased overall acceptability	[159]
Bread	Date palm fruit pulp	Replacement at 0, 25, 50, 75, and 100% of sugar	Increased the nutritional value (higher protein, fiber and ash content, and decrease in the level carbohydrate content)	[160]
Bread	Date palm fruit flour	Replacement at 0, 50 and 100% of sugar	Higher essential nutrients with many potential health benefits (increased protein, fiber, ash, vitamin and minerals)	[161]
Fortified bread	Date paste	15, 25, 35%	Improved the nutrient composition, storage stability, physical and sensory properties of bread	[162]
Cereal flakes	Date syrup	25, 50, 75 and 100	Acceptable to consumers, Improved nutrient values and potential health benefits	[166]
Candy	Date palm 10%	0–10%	Improved in the nutritional properties, the functional, phytochemical, and antioxidant properties and decreasing starch content	[151]
Snack Date bar	Date and date syrup	30 and 60% date and 20% syrup	Higher fracturability, fiber, ash, Ca, K, Mg, Fe with 60% date	[136]
Snack bar	Date paste	40, 50, 60 and 70%	Higher fiber. Improved the technological qualities 50% date paste were the formulation with the best sensory characteristics	[165]
Date bars	Date paste from immature fruits	100%	High organoleptic acceptability as well as microbial safety up to 30 days at room temperature and 50 days under refrigeration	[162]
Date-based bars	Date paste and date syrup	50% date paste and 6.5 date syrup	Higher ash, crude fiber, Ca, Cu, Fe, Zn, Mn, and Se, Lysine, Methionine, Histidine, Threonine, Phenylalanine, Isoleucine, and Cystine Better sensory evaluation	[169]
Original beer (nonalcohol)	Bleached date syrup	25, 50, 75 and 100%	The sample with 50% date syrup stands to be acceptable having maintained a Improved the physical characteristics	[109]
Fermented whey beverage	Date syrup	10, 12.5 and 15%	12.5% higher physicochemical, microbial and sensory properties	[150]

Color Indices (L*, a*, b*).

## 7. Conclusions

Rising cases of obesity and diabetes, coupled with the cardiometabolic risks linked to high sugar consumption, pose a major challenge to the food industry. To address this problem, there is an urgent need for the industry to advocate and facilitate improvements in the nutritional composition of processed products, particularly in terms of sugar type and content. Natural sources of sugar offer not only sweetness but also additional nutritional value, which can protect against certain diseases rather than simply providing empty calories. One promising approach is to replace added sugar with natural alternatives, such as dates, thus introducing a novel strategy to develop healthier foods by providing the food product with the macro- and micronutrients of dates in addition to sweetness.

Products such as fruit bars, dairy products and bread can be reformulated with dates, eliminating the need for additional sugar. This transformation makes them alternative consumption options in both high and low season, enriched by incorporating an undervalued, locally or regionally sourced product into their composition. From a nutritional point of view, this substitution of empty sugars by the sweet fruits of the date palm, which offer high levels of bioactive components such as fiber, polyphenols and minerals such as potassium, thus conferring important health benefits on consumers. This review suggests utilizing damaged dates and by-products as sugar substitutes in sweet food processing, offering health benefits and supporting sustainable practices. This approach efficiently uses discarded resources, aligning with circular economy principles for environmentally friendly production.

## Figures and Tables

**Table 1:** Brief overview of the main natural-nutritive sweeteners. Information recovered from [27,68] and the side references found in these works.

Sweetener	Composition	Properties	Applicability
Honey	60–85% sugars (mainly glucose and fructose, and 1–15% sucrose), 12–23% water, minerals, vitamins, bioactive compounds (phenolics)	Glycemic index: 54–59. Antioxidant, antimicrobial, anti-inflammatory.	Wide human consumption, nutritional and therapeutic applications. Drug in-home treatment for infections and burns
Molasses	30–40% sucrose, 4–9% glucose, 5–12% fructose 17–25% water, phenolic compounds, and traces of amino acids and vitamins	Glycemic index: 55. Humectant and colligative. Auto-immune, auto-inflammatory and antioxidant.	Food processing (e.g., masker undesired flavor). Coloring agent to improve visual presentation in baked foods
Maple syrup	60–65% sucrose, and other sugars as xylose, glucose or arabinose. Organic acids, amino acids minerals and phenolic compounds	Glycemic index: 54–65. Antioxidant, anti-mutagenic and antiproliferative in human cancer. Benefits in type 2 diabetes and Alzheimer’s diseases	Condiment for bakery products, in craft soda, or formulator in some beverage and snacks
Agave nectar	Approximately 90% fructose. Inulin and polyphenols	Low-glycemic index (17–27). Suitable for obesity and diabetes prevention. Boosted metabolic system, anti-obesity, anti-aging, chemoprotective and immunomodulatory effects	Sugar substitute in a wide range of foodstuffs: cheese, cookies, bread, cereal bar snacks, chocolate, guava purees, ice cream, sport drinks or yogurts
Coconut sugar	75% sucrose, < 25% fructose. High vitamin and minerals content	Glycemic index: 35–40	Additive in cakes, cookies, parfaits or sauces, sprinkled on top of granola
Palm sugar	91% sugar and 6% reduced sugars. Significant concentration of vitamins, minerals and phenolics	Glycemic index: 70 Cytoprotective activity against NIH3T3 fibroblast cells and cell proliferation (antioxidative agent)	Food additive in sweet soy sauce, desserts or beverages
**Sorghum syrup**	69% sugar (11% glucose, 6% fructose). Phenolic compounds, carotenoids, proteins and vitamins	Anticancer and anti-obesity effects. Preventive for cardiovascular diseases	Beverages and food industries

## Data Availability

Not applicable.
